# Supplementary material for: SyNDI: synchronous network data integration framework
Source: BMC Bioinformatics. 2018 Nov 6;19:403. doi: 10.1186/s12859-018-2426-5 (PMC6219086; doi:10.1186/s12859-018-2426-5)
Supplement: Supplementary file 5 — Files of the example presented in Additional file 4. This additional file is a zip package that contains all files used in Additional file 4. Detailed descriptions of each of these files are presented in a README file included in this zip package. (GZ 30411 kb) [file 12859_2018_2426_MOESM5_ESM.gz › SyNDITools/Meme2Fimo/meme_4.9.0_4.tar.gz!/meme_4.9.0/website/html/mhmm-options.in]

Meta-MEME v3.3


> ### Advanced options
>
> In the course of building and using a motif-based hidden Markov
> model, Meta-MEME requires that various parameters be set. By
> default, Meta-MEME employs reasonable values for these parameters.
> However, when submitting a job to the server, you may choose to
> override the default settings. The following list explains each
> parameter and gives its default value.
>
> ### Model building
>
> - **Model topology**  
>   By default, Meta-MEME builds models that have a linear topology, in
>   which the motifs are arranged like beads on a string. If, however,
>   you believe that the family you are interested in contains repeated
>   or shuffled elements, you may wish to build a model in which every
>   motif is connected to every other motif. Note that completely
>   connected models cannot be used to create multiple alignments.
>
> - **Motifs**  
>   By default, Meta-MEME includes in the hidden Markov model all the
>   motifs that appear in more than half of the sequences in the
>   training set. You may choose instead to include more or fewer of
>   the given motifs.
> - **Spacer models**  
>   Meta-MEME models the non-motif, spacer regions of the given family
>   using HMM states with self-loops on them. Because a single such
>   state yields an exponential distribution of spacer lengths,
>   Meta-MEME can use several such states in succession to give a more
>   bell-shaped distribution of spacer lengths. Note that these
>   multi-state spacer models are only useful in conjunction with total
>   probability scoring.
>
> ### Homology detection
>
> - **Scoring**  
>   By default, when Meta-MEME uses a model to search a sequence
>   database for homologs, the sequences are scored using the Viterbi
>   algorithm. This algorithm computes the most likely path through the
>   model, given the sequence. Alternatively, you can ask Meta-MEME to
>   compute the total probability of the sequence with respect to all
>   paths through the model.
> - **Score threshold**  
>   Meta-MEME reports homology log-odds scores in bits. Typically,
>   Meta-MEME only reports scores for sequences that score above zero.
>   You can set the score reporting threshold to some other value. Note
>   that, regardless of the threshold you select, the Meta-MEME server
>   will report at least 10 scores and at most 1000 scores.
> - **Minimum number of sequences**  
>   This parameter determines the minimum number of homology scores
>   reported by Meta-MEME. The default value is 10.
> - **Maximum number of sequences**  
>   This parameter determines the maximum number of homology scores
>   reported by Meta-MEME. The default value is 100.
>
> ---
>
> Return to the Meta-MEME home page.
>
> *Please send comments and questions to Charles E. Grant at
> @METAMEME\_CONTACT@.*
